# Measuring inconsistencies can lead you forward: Imageability and the x-ception theory

**DOI:** 10.3389/fpsyg.2014.00708

**Published:** 2014-07-15

**Authors:** Sara Dellantonio, Claudio Mulatti, Luigi Pastore, Remo Job

**Affiliations:** ^1^Psychology and Cognitive Science, Università degli Studi di TrentoTrento, Italy; ^2^Department of Developmental Psychology and Socialisation, Università degli Studi di PadovaPadova, Italy; ^3^Department of Educational Sciences, Psychology, Communication, Università degli Studi di BariBari, Italy

**Keywords:** imageability, concreteness, abstract words, concrete words, emotion words

## Abstract

According to the traditional view, both imageability and concreteness ratings reflect the way word meanings rely on information mediated by the senses. As a consequence, the two measures should and do correlate. The link between these two indexes was already hypothesized and demonstrated by Paivio et al. (1968) in a seminal article, where they introduced the idea of imageability ratings for the first time. However, in this first study, they also noted a contrasting pattern in the ratings for imageability and concreteness with some words that refer to affective attitudes or emotional states receiving high imageability but low concreteness ratings. Recent studies confirm this inconsistency (e.g., Altarriba and Bauer, 2004) leading to the claim that emotion words form a particular class of terms different from both concrete and abstract words. Here we use the MRC psycholinguistic database to show that the there are other classes of terms for which imageability and concreteness are uncorrelated. We show that the common feature of these word classes is that they directly or indirectly refer to proprioceptive, interoceptive, or affective states, i.e., to internal, body-related, sensory experiences. Thus, imageability and concreteness can no longer be considered interchangeable constructs; rather, imageability is a different, and perhaps more interesting, measure: it not only reflects the ease with which memories of external events come to mind, as previously hypothesized, but also reflects the ease with which memories of internal events come to mind.

## Introduction

Analogously to frequency and familiarity, concreteness, and imageability are properties of words (referents) that are partially intertwined. According to the traditional view, both imageability and concreteness ratings reflect the way word meanings rely on information mediated by the senses. For this reason, they are the most relevant operational constructs to address the question of the processing differences between concrete and abstract words, i.e., between words denoting things that can be perceived by the senses and words that do not have this kind of reference. Since they are hypothesized to detect analogous properties, the two measures should strongly correlate, as indeed they do. And because of this strong correlation, in experiments for the selection of concrete vs. abstract verbal material they are often used interchangeably (see e.g., Reilly and Kean, 2007; Connell and Lynott, 2012).

The link between these two indexes was already hypothesized and demonstrated by Paivio et al. (1968), who introduced the idea of imageability ratings for the first time. However, already in this study a contrasting pattern was reported and has not yet been fully accounted for: a number of words referring mainly to affective attitudes or emotional states received high imageability ratings but low concreteness ratings. Recent studies confirm this anomaly (e.g., Altarriba et al., 1999; Wiemer-Hastings et al., 2001; Altarriba and Bauer, 2004; Wiemer-Hastings and Xu, 2005) and maintain that emotion words form a particular class of terms different from both concrete and abstract terms.

In this paper we present an account of this presumed inconsistency arguing that imageability ratings measure not only whether (how much) words rely on external sensory information, but also whether (how much) words rely on *internal bodily-related sensory experience*. Since imageability ratings are a joint measure of the link between word meanings and both external and internal sensory experience, while concreteness ratings measure the link to external sensory information only, we suggest that an index of the “weight” internal sensory information has with respect to the meaning of a word can be obtained by subtracting the concreteness rating of this word from its imageability rating.

To support this view we use the MRC psycholinguistic database (Coltheart, 1981; Wilson, 1988). First of all, on the basis of an analysis of the instructions used when collecting the imageability ratings included in the database, we suggest that the contrasting pattern observed with respect to these word classes is due to the fact that imageability ratings do not only reflect the ease/difficulty with which people can evoke a mental picture of the instances denoted by the word, as it commonly assumed (e.g., Vigliocco et al., 2009; Connell and Lynott, 2012). These instructions might rather have biased people to assign their imageability ratings on the basis of the ease/difficulty with which a word arouses sensory experience of any kind, including internal, body-related sensations. Secondly, we analyze the imageability and concreteness ratings of specific words included in these databases to show that, in addition to emotion words, there are also other classes of terms for which imageability and concreteness are uncorrelated and that the common feature of all kinds of words exhibiting this contrasting pattern is that they directly or indirectly refer to proprioceptive, interoceptive, or affective states, i.e., to internal, body-related, sensory experiences.

This shows that imageability and concreteness are not interchangeable constructs: imagery ratings are not only another means to assess the degree of concreteness of a word, but they are also a different, and perhaps more interesting, measure of the link between a word and some internal information pertaining to the state the word denotes. Since imageability ratings are a joint measure of the connections between words and both external and internal sensory experience, subtracting concreteness from imageability gives us a tangible measure of the internal sensory information aroused by a word. Even though this measure cannot be completely accurate since it is a result of ambiguous norming instructions, it can still indicate whether a word relies (more or less heavily) on body-related sensory information. Clearly, new collections of ratings on the basis of less ambiguous instructions are required in order to have more precise imageability ratings to use for experimentation. However, our study indicates a way to interpret the imageability construct from a new and possibly more fruitful perspective which allows us to both avoid the incongruities of the old measure and to assess more clearly in what respects concreteness and imageability converge and in what respects they instead diverge.

## Imageability in the MRC psycholinguistic database

Concrete (CONC) ratings for the MRC psycholinguistic databases were originally collected by Spreen and Schulz (1966). With the exception of the most recent database of 2013 (Brysbaert et al., 2013), later collections are included in the MRC database and rely on the same definition of concreteness and on the same instructions. As Spreen and Schulz (1966, p. 459) point out, starting from the twenties it became clear that there are differences in remembering, recognizing and understanding concrete and abstract verbal material. For the study of these differences they tried to work out a precise scale for concrete and abstract words, based on a non-ambiguous definition of the two poles. In fact, previous definitions interpreted the opposition of abstractness and concreteness in at least two different ways: on the one hand, in terms of general, i.e., generic vs. specific, and on the other, in terms of a difference in the nature of the referents of abstract and concrete terms–the referents of concrete terms are directly connected to sensory experience, while those of abstract words lack this connection. Spreen and Schulz (1966) opted for this last definition and suggested that the scale should measure whether the referents of a word can or cannot be experienced by the senses.

The imageability (IMAG) scale was first introduced by Paivio et al. (1968) as a further measure in addition to CONC to investigate psychological effects of linguistic abstractness-concreteness: in the context of their study, imagery is postulated to be “the major effective psychological attribute underlying abstractness-concreteness” (p. 2). High and low imagery ratings measure the ease or difficulty with which words arouse sensory images. These sensory images are defined in a rather vague manner as any kind of sensory experience evoked by words by recalling non-verbal representations of their referent. Concreteness is considered to be determined independently from imagery; however, highly concrete words are assumed to have a high image-arousing value, since they are particularly effective in evoking sensory images of their referents which, in this case, consist mainly in mental pictures of them.

The correlation between IMAG and CONC was confirmed by Paivio et al. (1968) on 925 words. This correlation provided evidence for Paivio's *Dual Code Theory* (Paivio, 1971, 1986, 2007), according to which cognitive processing is carried out on the basis of two different subsystems, “one specialized for the representation and processing of information concerning nonverbal objects and events, the other specialized for dealing with language (Paivio, 1986, p. 53). The nonverbal (symbolic) subsystem is referred to as the imagery system: “its critical functions include the analysis of scenes and the generation of mental images (both functions encompassing other sensory modalities in addition to visual)” (Paivio, 1986, pp. 53–54). The language-specialized system is called the verbal system. In Paivio's perspective, the nonverbal system of imagery is activated primarily by concrete (i.e., perceivable) stimuli (Paivio, 1986, p. 68), therefore words with a high CONC rating should have high IMAG ratings, while words with a low CONC ratings (i.e., abstract words) should have low IMAG ratings. According to Paivio, this dual system can also account for the so-called “concreteness effect,” i.e., the fact that concrete words are processed more easily and quickly than abstract words because, while abstract words activate verbal representations only, concrete words activate representations in both the verbal and in the imagery system, and this facilitates the referential act.

The idea that CONC and IMAG are strongly correlated both theoretically and from the point of view of their ratings has become established in the literature (Paivio et al., 1968 found a correlation of 0.83; this correlation has been confirmed using larger word numbers by several other studies that report values ranging from 0.64 to 0.95: see e.g., Christian et al., 1978; Toglia and Battig, 1978; Gilhooly and Logie, 1980; Rubin, 1980; Friendly et al., 1982; Rubin and Friendly, 1986; Schwanenflugel et al., 1988; Benjafield and Muckenheim, 1989). This is the reason why these two measures are considered interchangeable and are both used to study the processing differences between abstract and concrete verbal material. As e.g., Reilly and Kean (2007) point out: “Although imageability and concreteness are technically different psycholinguistic constructs, the correlation between these variables is so strong that many authors use the terms interchangeably. Here we make the same assumption of synonymy between imageability and concreteness in terms of theory (i.e., concreteness effects—imageability effects)” (p. 158). The same point has been made more recently by Connell and Lynott (2012): “Imageability ratings are frequently used interchangeably with concreteness ratings in the experimental literature […] because of their high correlation and theoretical relationship in dual coding theory” (p. 453).

However, even though the correlation between CONC and IMAG is quite strong, (a) it is lower than expected and (b) exhibits some relevant anomalies. As Paivio and colleagues underline: “the correlation between I and C, although substantial, is not as high as one might expect if it is assumed that both scales measure the same underlying variable” (Paivio et al., 1968, p. 7). According to Paivio et al. (1968) the problem is due to some sets of problematic items whose IMAG ratings are significantly greater than their CONC ratings^1^. As they note, these items exhibit an interesting semantic similarity: “Most of these are words with strong emotional and evaluative connotations. The largest group consists of terms referring to affective reactions or affective attitudes” (Paivio et al., 1968, p. 7).

Paivio et al. (1968) do not offer any explanation for the contrasting patterns for IMAG and CONC. However they suggest that also in these cases high IMAG must be due to the fact that the word easily evokes some kind of sensory experience, which in this case seems to be of an affective kind: “These words appear to have the common property of having been associated with sensory experience (usually affective in nature)” (p. 7). These observations open the door for the hypothesis also embraced by Paivio in his later work (1986, p. 79) that affective and emotional words have a high IMAG rating because they directly evoke the sensory experience of an affective arousal.

Similar inconsistencies are also pointed out by recent studies that interpret them in a way analogous to Paivio's suggesting that emotion words are different from both abstract and concrete words as regards their CONC and IMAG ratings and must therefore be considered as a particular word class with specific characteristics (Altarriba et al., 1999; Altarriba and Bauer, 2004; Wiemer-Hastings and Xu, 2005). In particular, as the results of Altrarriba's study (2004) indicate: “concepts represented by emotion words are more imageable and are easier to of a context for than abstract words but are less concrete than abstract words. They are less imageable, less concrete, and less likely to activate a context than concrete words” (p. 407). Therefore, even though emotion words are, as one would expect, less CONC than concrete words, they turn out to be also less CONC than abstract words, even though their IMAG is significantly greater than that of abstract words. Thus, for this word class the divergence between IMAG and CONC is particularly broad. As a matter of fact, our analysis of the IMAG and CONC ratings included in the MRC psycholinguistic database, which has been the main source for these measures showed that the difference between IMAG and CONC is significantly greater for emotion terms than for any other randomly chosen control group of words (we will come back to this aspect in the next section).

Altarriba et al. (1999), Altarriba and Bauer (2004) emphasize the fact that examining the unique qualities of emotion words with respect to other classes of terms is particularly important since it helps us understand how people recognize and label emotions. However, we think that the uniqueness of the IMAG and CONC ratings for emotion words can also help clarify the linguistic construct of imageability which is often considered vague and subject to different interpretations (e.g., Connell and Lynott, 2012; Westbury et al., 2013; Dellantonio et al., 2014). In fact, the anomaly of the IMAG and CONC ratings in the case of emotion words can be explained only by specifying what precisely IMAG measures and what is the specific difference between the constructs of IMAG and CONC. The key point to disentangle in this respect lies first of all in the content of the instructions given to subjects for assigning the CONC and the IMAG ratings included in the MRC database.

## Instruction-bound ratings?

The original instructions for concreteness ratings were developed by Spreen and Schulz (1966), and then used in almost the same form by Paivio et al. (1968): however, while Spreen and Schulz (1966) labeled the end-points of the rating scales “low concreteness” and “high concreteness,” Paivio et al. (1968) labeled them “high concreteness” and “high abstractness.” Later collections used either the one or the other label interchangeably.

Spreen and Schulz's (1966) instructions for concreteness were: “Nouns may refer to persons, places, and things that can be seen, heard, felt, smelled, or tasted or to more abstract concepts that cannot be experienced by our senses. The purpose of this experiment is to rate a list of words with respect to “concreteness” in term of sense-experience. Any word that refers to objects, material or persons should receive a high concreteness rating; any word that refers to an abstract concept that cannot be experienced by the senses should receive a low concreteness rating. Think of the words “chair” and “independence.” “Chair” can be experienced by our senses and therefore should be rated as high concrete; “independence” cannot be experienced by the senses as such and therefore should be rated as low concrete (abstract)” (p. 460).

The original instructions for imageability ratings were developed by Paivio et al. (1968) and were the following: “Nouns differ in their capacity to arouse mental images of things or events. Some words arouse a sensory experience, such as a mental picture or sound, very quickly and easily, whereas others may do so only with difficulty (i.e., after a long delay) or not at all. The purpose of this experiment is to rate a list of words as to the ease or difficulty with which they arouse mental images. Any word which, in your estimation, arouses a mental image (i.e., a mental picture, or sound, or other sensory experience) very quickly and easily should be given a high imagery rating: any word that arouses a mental image with difficulty or not at all should be given a low imagery rating. Think of the words “apple” or “fact.” Apple would probably arouse an image relatively easily and would be rated as high imagery; fact would probably do so with difficulty and would be rated as low imagery” (p. 4).

Both sets of instruction bias toward the sense of vision. According to the concreteness instructions, something is concrete if it can be perceived through (at least one of) the senses. However, as it is has been already pointed out (Connell and Lynott, 2012, p. 461), the examples mentioned in the second part of the definition (“objects, material or persons” as well as “chair” vs. “independence”) might have biased people to rely for their ratings (also) on a different idea of concreteness which resembles more closely the everyday understanding of the word “concrete” and its dictionary definition, according to which “concrete” means material or physical and an object is concrete only if it has a material composition. Since material objects are perceived mainly or primarily through vision, people's ratings probably favored this sense over the others. Analogously to the instructions for concreteness, the instruction for imageability also evoked an idea of imageability that is primarily visual and related to the ease/difficulty with which people can form a mental picture of the referent of a word. Moreover, even though in Paivio's view “mental images” describe traces stored in memory of all kind of sensations, the term “image” recalls quite strongly the idea of a visual picture. Thus, for this aspect IMAG ratings follows criteria that overlap that of concreteness, since the instances people can more easily form a mental picture of are external, material things that they can see.

However, despite what some studies maintain (e.g., Vigliocco et al., 2009; Connell and Lynott, 2012), this is not the only relevant aspect IMAG measures. Just as CONC also measures whether/in what degree the referents of words can be experienced by senses other than sight, so IMAG measures also whether/in what degree a word arouses other kinds of sensory experience. More specifically, the request to estimate IMAG depending on whether/how much a word arouses “sensory experience” without further specifications might have lead participants to assign their ratings on the basis of the ease/difficulty with which words arouse *any kind* of sensory experience stored in memory, including internal, body-related sensations. Following Paivio et al. (1968), Paivio (1986) and Vigliocco et al. (2009), we propose that affective arousal is a kind of sensory experience, based on internal feeling rather than derived from the external senses.

## A new hypothesis: looking at the inconsistencies from an “internal” perspective

This idea that word meaning might rely jointly on both internal and external sensory experience suggests that IMAG ratings might also track—at least in part—the internal and bodily-related sensory experience evoked by words. If so, IMAG diverges from CONC, and becomes a different, and more interesting measure of both the external and the internal experiential grounding of words. Since in our interpretation the imageability measure is a result of ambiguous norming instructions that lead people to assign ratings relying on their commonsense notion of sensory information, as including both internal and external information sources rather than solely external ones, we cannot assume that it is perfectly accurate. However, if we assume that people do not rely only on visual information to provide the ratings, but also spontaneously took into account their internal sensory experience and thus assigned a certain degree of IMAG to all words that aroused external and/or internal sensory experiences, then we can account for the divergence between IMAG and CONC in the case of emotion words.

If this hypothesis is correct, the class of emotion words should not be the only terminological class exhibiting a significant divergence between IMAG and CONC. In fact, all words that give rise to some kind of internal sensory experience should have an IMAG rating that is significantly higher than the CONC rating. The more a word arouses internal sensory experience, the greater should be the divergence between IMAG and CONC.

A word class that resembles emotion words insofar as it denotes body-related conditions which are experienced internally is that class denoting proprioceptive and interoceptive states. Proprioception and interoception are closely related notions: proprioception indicates our aware experience of the position of our body (see e.g., Berthoz, 2000); while interoception describe people's general conscious experience of their bodily states or of specific conditions of parts of their body (Craig, 2003, 2009, 2010). Words describing typical proprioceptive states and interoceptive states are e.g., balance, relaxation, movement, tremor, sit, rest, jump, run, walk etc. on the one hand and on the other ache, sick, hunger, thirsty, warmth, itch, pain, cold, etc.

Emotion, proprioceptive, and interoceptive words might however not be the only ones relying on internal, bodily-related sensory experience. In fact, some recent studies carried out in the field of so called embodied cognition suggest that abstract words are also grounded in internal states, especially affective and mental states (see e.g., Barsalou and Wiemer-Hastings, 2005; Kousta et al., 2009, 2011; Vigliocco et al., 2009; for a review of older studies see e.g., Barsalou, 1999, p. 599). In particular, the studies by Wiemer-Hastings et al. show that abstract words tend to have more introspective and affective associations than concrete words (Wiemer-Hastings and Xu, 2005; Vigliocco et al., 2009; Kousta et al., 2011). As these studies suggest, abstract concepts clearly cannot rely only on affective information, their representation must also be based on linguistic information, and the exact proportion of affective and linguistic information will vary depending on the word (see e.g., Vigliocco et al., 2009; Kousta et al., 2011). However, if we admit that abstract words do indeed also rely at least minimally on internal sensory experience and hypothesize that IMAG ratings measure whether a word arouses internal sensory experience, then in the case of abstract words the correlation between CONC and IMAG should be significantly smaller than in the case of concrete words because IMAG ratings should be relatively higher than CONC ratings.

Some results in line with this prediction were already reported by Altarriba et al. (1999) and by Wiemer-Hastings et al. (2001); however a more accurate analysis is needed. According to our hypothesis, correlation patterns should differ when calculated separately for decreasing CONC ratings: the more abstract a word is, the weaker the correlation between CONC and IMAG should become. In addition, since the proportion of affective and linguistic information abstract words rely on varies depending on the kind of words we are considering, we expect that highly theoretical words with a technical meaning that have only a limited everyday use and strictly depend on their linguistic definition (e.g., adverb, literal, plenipotentiary, causality, regulation, abduction, deduction, axiom, factor, fallacy, function, suffrage etc.) will have relatively low IMAG ratings with respect to CONC ratings. More specifically, since their proportion of linguistic information is particularly high in comparison to sensory information, if our hypothesis about IMAG is correct, the difference between the IMAG ratings and the CONC ratings for this class of words should be either smaller than, or comparable to, that of other word groups.

## Testing the hypothesis

To prove our hypothesis, we analyzed the CONC and the IMAG ratings included in the MRC database, which is an important source for these measures in psycholinguistic studies and constitutes the only database available in which CONC and IMAG were collected simultaneously by the same studies. These not only rely on exactly the same instruction we discussed previously, but were also driven by the intent of understanding the relationship between IMAG and CONC.

Later collection of IMAG and CONC ratings available in English are not directly relevant with respect to our hypothesis for a number of reasons. First of all, recent collections of IMAG ratings do not also include CONC ratings (Bird et al., 2001; Cortese and Fugett, 2004; Stadthagen-Gonzalez and Davis, 2006; Schock et al., 2012). Since the clue to understand the theoretical peculiarities of the construct of imageability resides in the anomalies with respect to concreteness, it is by considering the imageability ratings in relation to the concreteness ratings—i.e., by comparing them—that a new insight into the construct of imageability can be achieved. Secondly, some collections rely on instructions that differ at least in some respect from the one we discussed: this is the case for the recently published database of CONC (Brysbaert et al., 2013) as well as for the collection of IMAG ratings carried out by Bird et al. (2001)^2^. Thirdly, some of these collections are very specific in scope and consider only monosyllabic and disyllabic words (Cortese and Fugett, 2004; Schock et al., 2012). Finally, Stadthagen-Gonzalez's and Davis' database is obtained merging their data with Gilhooly and Logie's (1980) collection, which is already included in the MRC database.

The MRC psycholinguistic database includes 9240 words possessing an IMAG rating and 8228 words possessing a CONC rating. Both are derived from merging three sets of norms: the Colorado Norms (Toglia and Battig, 1978), the Pavio Norms (unpublished, these are an expansion of the norms of Paivio et al., 1968), and the Gilhooly-Logie norms (Gilhooly and Logie, 1980). A large part of the data from Toglia and Battig (1978) was validated by Cortese and Fugett (2004). The values are in the range 100–700. Words are partitioned in ten syntactic categories: nouns, adjectives, verbs, adverbs, conjunctions, pronouns, interjections, past participles, other.

### Selection of stimuli

For our analysis we considered only words that have both an IMAG and a CONC rating and we excluded conjunctions, pronouns, interjections, and the class labeled “other.” Repetitions were also excluded. This leaves 4260 words.

Across all words, mean IMAG and CONC ratings are 456.4 and 438.7 respectively. The correlation between IMAG and CONC is significant (*r* = 0.835, *p* < 0.001), which demonstrates—as has been previously observed—that the two constructs are tightly interconnected. Interestingly, if two groups of words are construed as a function of CONC (low vs. high CONC ratings, 2130 words in each group; mean CONC and IMAG ratings for the low CONC group: 331.6 and 376.6, respectively; mean CONC and IMAG ratings for the high CONC group: 545.8 and 536.3, respectively), the correlation between IMAG and CONC for the low CONC group (*r* = 0.550, *p* < 0.001) is significantly smaller than the correlation between IMAG and CONC for the high CONC group (*r* = 0.661, *p* < 0.001), *z* = 5.7, *p* < 0.001^3^. This is compatible with the view that IMAG ratings are less dependent upon CONC ratings for the abstract (i.e., low concrete) words with respect to the concrete words. Since, as specified in section Instruction-Bound Ratings?, abstract words generally rely on more introspective information than concrete words, these different correlation patterns suggest that IMAG does not entirely depend on CONC, but it is also a measure of something else, and specifically of the ease/difficulty with which a word evokes internal sensory experience of any kind (be it e.g., emotional, proprioceptive or interoceptive).

To test the hypothesis that IMAG ratings depend on the ease/difficulty with which a word arouses both external and/or internal sensory experience, and that a discrepancy between CONC and IMAG may be diagnostic of the relative contribution of the two kinds of sensory information, we selected three groups of words: (i) 36 emotional words (whose anomalous behavior as for their IMAG and CONC ratings has already been singled out by other studies—on this point see section Imageability in the MRC Psycholinguistic Database), (ii) 56 proprioceptive or interoceptive words (which we call globally *X-ceptive* to indicate that the same considerations we develop for proprioception and interoception should apply for any kind of states based on an internal perception), and (iii) 110 theoretical terms (i.e., abstract technical terms whose meaning is not grounded on internal states, but depends rather on a linguistic definition given in the framework of a theory). In addition, we construed ten control groups of 100 randomly selected words to compare with (i), (ii), and (iii). Selection of the words to serve in the control groups was accomplished through a computerized algorithm, with the only restriction that none of the words in the emotional, X-ceptive or theoretical group could be selected to serve in the control groups.

(i) The class of emotion words combines two kinds of words: those strictly denoting emotions and those denoting what are more correctly called moods (or background feelings—e.g., depression, anxiety, wellness, distress, etc.). In order to individuate a particularly salient and unambiguous set of terms, our selection from the MRC database was based on the emotions/moods described by a number of studies (which sometimes consider a mixture of the two). As for emotions, we included only emotions considered as basic (Tomkins, 1962, 1963; Ekman et al., 1969; Plutchik, 1980; Ekman, 1999; Reizenzein, 2009; Kassam et al., 2013). While the words denoting basic emotions are all strictly derived from the mentioned studies, the list of the words denoting moods is more freely composed starting from the examples and the definitions given in various studies (Ekman, 1994; Damasio, 1999; Prinz, 2004). Emotion words strongly rely on internal affective experience; thus, if IMAG ratings measure how easily a word evokes not only external but also internal sensory information, IMAG ratings for this class of words should be significantly higher than CONC ratings compared to the other groups of words.

(ii) Words denoting proprioceptive and interoceptive (X-ceptive) states were selected from the MRC database starting from the examples considered in the studies of Berthoz (2000) and Craig (2003, 2009, 2010). Since proprioceptive and interoceptive (X-ceptive) states are analogous to emotions due to the fact that they are based on internal sensory experience, we expect words denoting these states to behave like emotion words and exhibit IMAG ratings significantly higher than CONC ratings with respect to other groups of words.

iii) For the selection of theoretical words we could not rely on previous studies, even though the definition of a class of theoretical terms as opposed to a class of observational terms was already introduced by Paivio (Paivio, 1986, p. 10 Clark and Paivio, 1989). However, while Paivio interpreted theoretical terms simply as abstract terms, we consider theoretical terms as an autonomous subclass of abstract words. In our account, theoretical terms are technical words with a definitional structure whose meaning is fixed in the framework of a theory. We identified this group of words one by one in the database according to this criterion: the chosen terms belong to the technical jargon of a discipline and therefore strictly depend on a specific linguistic definition. Thus, we avoided terms that denote anything that can be perceived through the senses. An example is the mathematical term “axiom,” i.e., a statement or formula on which an abstractly defined structure is based. Other than from mathematics, terms come from physics (e.g., “causality”), linguistics (e.g., “conjugation”), politics and law (e.g., “legislation”), logic (e.g., “deduction”), and science in general (e.g., “theory”). Since this class should rely only very weakly on internal sensory experience, will have relatively low IMAG ratings with respect to CONC ratings. Specifically, we expect that the difference between the IMAG ratings and the CONC ratings for this class of words will be either smaller than, or comparable to, that of other word groups (control groups as well as emotion and X-ception words).

### Procedure

We compared the differences between ratings of IMAG and CONC of these three groups against the differences between the ratings of IMAG and CONC of ten control groups including 100 randomly selected words (basically, a bootstrap). The idea here is that the mean differences between IMAG and CONC of the control groups—being composed of randomly selected words—reflect the mean differences between IMAG and CONC of the population they derived from. Therefore, if one (or more) of the three experimental groups consistently and significantly differs from the control group(s), then we can conclude that that experimental group differs from the population on the tested dimensions. The comparisons were made using an ANOVA with Group [experimental (X-ception, emotion, or technical) vs. control (each of the 10 control groups)] as a between-items factor. In addition, each of the experimental groups was compared with the other two experimental groups.

### Results

The results are reported in Table 1. The first 10 rows refer to the comparison of each experimental group of words with one of the control groups. The last two rows refer to the comparison among the experimental groups of words.

**Table 1:**
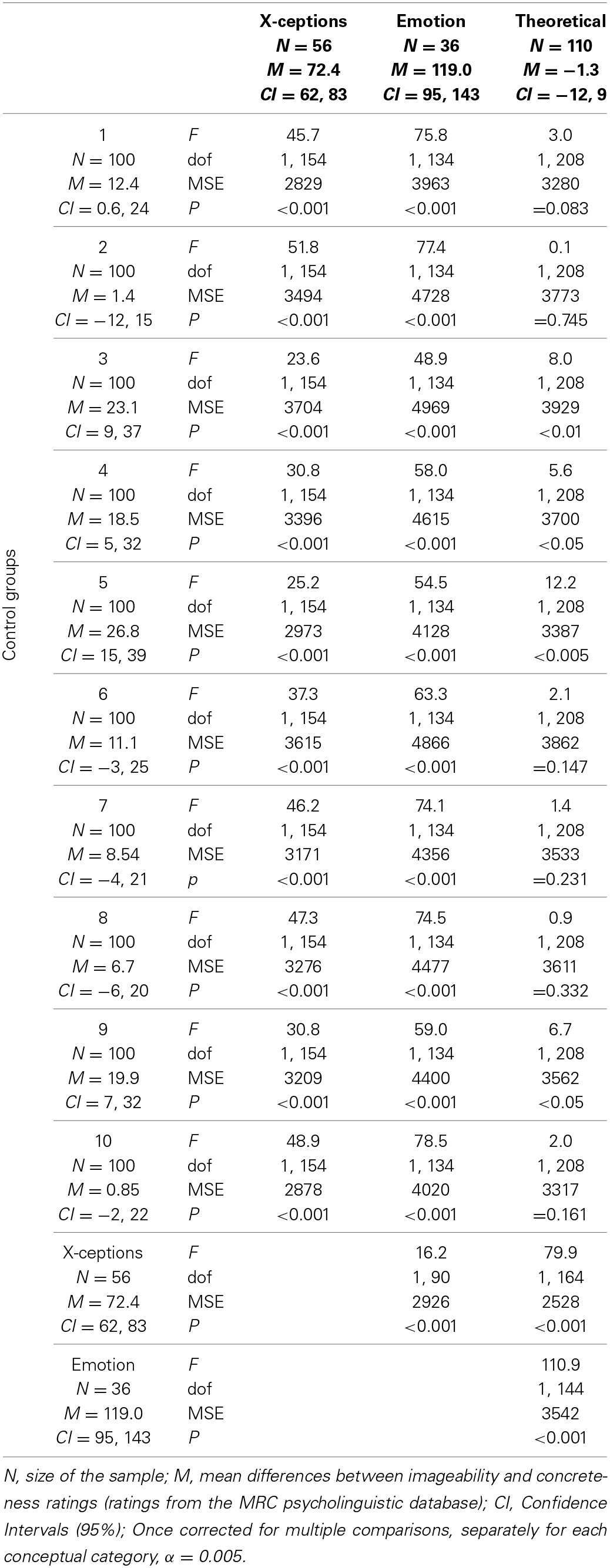
**Results of the ANOVAs**.

*N, size of the sample; M, mean differences between imageability and concreteness ratings (ratings from the MRC psycholinguistic database); CI, Confidence Intervals (95%); Once corrected for multiple comparisons, separately for each conceptual category, α = 0.005*.

As expected, the differences between IMAG and CONC for the X-ception words are significantly higher than those of the control groups. Also, and in line with previous evidence, the differences between IMAG and CONC for the emotion words are significantly higher than those of the control groups. In addition, and congruently with the theory at the basis of our hypothesis, the differences between IMAG and CONC for the theoretical/technical words are either smaller than, or comparable to, those of the control groups. Unexpectedly, the differences between IMAG and CONC of the X-ception words are significantly smaller than those of the emotion words: This will be dealt with in the General Discussion.

## General discussion

According to the hypothesis we put forward, IMAG is a construct based on two factors. On the one hand, IMAG depends on CONC, since it measures the ease/difficulty with which a word evokes external (mainly visual) sensory experience related to the objects it denotes. On the other hand, IMAG is partially independent of CONC and measures the ease/difficulty with which a word evokes internal sensory experience. To test this hypothesis we used the IMAG and CONC ratings included in the MRC database. Since we assume that IMAG is always linked to CONC and that IMAG ratings will therefore always reflect to a certain extent CONC ratings, we are not interested in analyzing IMAG and CONC ratings *per se*, but we focus on the difference between IMAG and CONC ratings which reveals a value of IMAG independent from CONC.

Our analysis started from some basic assumptions regarding what type of information different classes of words rely on. Indeed, words might be grounded on both external or internal sensory information: (a) while concrete words rely primarily on external sensory information, in general abstract words are mainly based, to a larger or smaller extent, on internal sensory information and on linguistic information. (b) Among the abstract terms that surely rely for a large part on internal sensory information there are words denoting emotions as well as proprioceptive and interoceptive states. (c) On the contrary, theoretical words denoting technical notions will probably be mainly linguistic constructs and be based only to a very small extent on sensory information of any kind.

(a) Moving from this premise, we examined first of all whether in general the correlation between CONC and IMAG varies for concrete and abstract words. While in the case of concrete words, IMAG ratings should be just a function of CONC ratings, in the case of abstract words which rely to a certain degree on internal information the correlation between CONC and IMAG should be significantly smaller. Our analysis on two groups of words constructed as a function of CONC confirmed this hypothesis.

(b) Secondly, we selected two groups of words from the database, one denoting emotions and the other proprioceptive/interoceptive (x-ceptive) states. Since these classes of words rely to a large degree on internal information, we expected that the difference between IMAG and CONC for both classes would be significantly higher than that for the control groups. Our statistical analysis supports this hypothesis.

An unexpected finding here is that the difference between IMAG and CONC for the X-ception words is significantly smaller than that for the emotion words. There are at least two possible explanations for this result.

First, one could speculate that it is due to the fact that emotions rely on internal bodily information in a twofold manner. On the one hand, an emotional state is revealed by a specific affective arousal and internal feelings. On the other hand, emotions bring about specific bodily reactions and above all specific facial expressions which are an essential part of emotions (see e.g., Ekman, 1984) and are recorded through interoception giving additional bodily information on the state. Thus, one could hypothesize that the higher difference between IMAG and CONC for emotion words compared with x-ceptive words is due to this double binding between emotions and internal sensory experience: in this case, mean IMAG ratings for emotion words should be higher than those for X-ception words, whereas mean CONC ratings for the two classes of words should be similar. A second possible explanation is that the sensory information corresponding to proprioceptive and interoceptive states (i.e., internal world perception) is “qualitatively comparable” to (external world-) perception and is therefore interpreted as more concrete than the affective arousal/feelings corresponding to emotions. That is, people could consider words like “ache,” “hunger,” “cold,” “hot,” “motion,” “itch” as denoting more tangible and specific (i.e., concrete) states than words like “happiness,” “sadness,” “excitement,” “humiliation,” “jealousy” etc. As a consequence, in this case CONC ratings of X-ceptive words should be higher than those of emotional words, whereas mean IMAG ratings for the two classes of words should be similar.

To distinguish between these two hypotheses, we performed two ANOVAs. In one analysis, we compared the CONC ratings of X-ception and emotion words. This analysis showed that the concreteness ratings of X-ception words was significantly higher than the concreteness ratings of emotion words (means: 391 vs. 314, respectively; [*F*_(1, 90)_ = 39.4, MSE = 3334, *p* < 0.001]. In the second analysis we compared the IMAG ratings of X-ception and emotion words. This second analysis showed that the imageability ratings of X-ception words was significantly higher than the imageability ratings of emotion words [464 vs. 433, respectively, *F*_(1, 90)_ = 7.2, MSE = 2904, *p* < 0.01]. Unfortunately, these analyses do not allow us to conclusively decide in favor of either of the two hypotheses put forward above, since both IMAG and CONC ratings are lower for emotion words with respect to X-ception words. It is worth noting that the difference between the mean CONC ratings of the two classes of words is larger than the difference between the mean IMAG ratings, and this, if anything, provides (weak) support for the second of our hypotheses.

(c) Finally, we selected from the database a group of theoretical/technical words which should only weakly rely on internal information and therefore have relatively low IMAG ratings with respect to CONC ratings. Congruently with this hypothesis, the differences between IMAG and CONC for the theoretical/technical words turned out to be either smaller (4 out of 10 comparisons are significant if α = 0.05; 1 out of 10 comparisons are significant once α is corrected for multiple comparisons^4^; c.f. Table 1) then or comparable to those of the control groups.

Taken together, these results show that IMAG is not simply an alternative way to measure concreteness, but, instead, that IMAG provides specific information and depends in part on the strength with which words evoke body-internal sensations. We think that this result is extremely useful, among other things, for better understanding how to use IMAG and CONC ratings for experimental research.

One of the main applications of these ratings is in studies that analyze the processing advantages of some classes of words over others; most famously, the processing advantages of concrete vs. abstract words (the so-called concreteness effect). In this case, our results suggest not only that the two ratings should not be used interchangeably, but they also indicate that—in addition to a concreteness effect—it might be possible to identify an effect specifically related to imageability (and more precisely to “the side” of imageability that does not depend on concreteness), which measures the ease/difficulty with which words evoke some kind of internal sensory information. In this case, processing advantages should be observed for both emotion and X-ceptive words.

## Concluding remarks

In this paper we analyzed the IMAG and CONC ratings included in the MRC Psycholinguistic Database. We started by presenting the results of some previous studies showing that—even though there is a strong correlation between measures of IMAG and CONC—some words with low CONC ratings (i.e., abstract words) exhibit a contrasting pattern and have relatively high IMAG ratings. Also on the basis of an analysis of the instructions given to subjects during the collection of the ratings, we hypothesized that IMAG is only in part connected to CONC; while to a certain extent is independent from it and measures something different: i.e., the ease/difficulty with which a word arouses any kind of sensory experience including internal, body-related sensations. In order to validate this position, we carried out several analyses of the IMAG and CONC ratings in the database, individuating different groups of words and considering for each the difference between IMAG and CONC. All the results are congruent with the initial hypotheses.

These results show that IMAG ratings depend at least on two factors: i.e., on the one hand, on whether a word denotes concrete external objects (and for this aspect IMAG directly relies on CONC) and on the other, on whether a word is grounded in any kind of internal, body-related sensations. As we showed, this is the case for words denoting e.g., emotions as well as proprioceptive and interoceptive states.

This conclusion serves not only to reaffirm at least to some degree the reliability of the IMAG measure, in spite of the well-known inconsistencies that characterize it, which we interpreted from an entirely new perspective, but it also has relevant consequences at least with respect to two different points. On the one hand, it challenges the widely shared idea that CONC and IMAG are interchangeable scales measuring one and the same thing. On the other hand, our analysis helps to clarify the IMAG construct and to specify what it exactly measures. This has direct implication e.g. for the debate on the relationship between abstract and concrete. According to Vigliocco, Kousta, and collaborators (Vigliocco et al., 2009, 2013; Kousta et al., 2011) the dichotomy between abstract and concrete words is only apparent, as word meanings rely in different proportions on perception, internal information, and linguistic information: thus, generally people call “concrete” the words with an higher proportion of perceptual information, while they call “abstract” the words relying primarily on internal and linguistic information. Since imageability ratings are a joint measure of the link of words with both external and internal sensory experience, we suggest that the extent of a word's connection with internal sensory information can be obtained by subtracting its concreteness rating from its imageability rating. If the interpretation of imageability we put forward is correct, then the intersection between imageability and concreteness can indeed give us a tangible (even though not completely accurate) measure of the internal sensory information aroused by a word.

Our analysis shows also that the imageability and even concreteness measures are complex and problematic constructs, whose ratings undergo biases that cannot be completely controlled. New collections of ratings on the basis of less ambiguous instructions are required in order to have more precise measures to use for experimentation, i.e., to show among other things whether an abstract word mainly rely on linguistic information and is therefore theoretical or whether also an abstract word is strongly grounded in internal information.

### Conflict of interest statement

The authors declare that the research was conducted in the absence of any commercial or financial relationships that could be construed as a potential conflict of interest.

## Footnotes

^1^There were also a few terms with high concreteness and low imageability ratings like e.g., “aster,” “astrolabe” or “stein,” but these are easy to explain: people know they denote concrete objects, but they do not know what they look like.

^2^The new instructions for concreteness are particularly problematic since they ask people to evaluate actions as something concrete equating them from a semantic point of view with objects. A study specifically devoted to the role of instructions in collecting useful psycholinguistic data on concreteness is in preparation.

^3^Correlation were compared according to the following procedure. First *r* values have been converted into *z* values (Fisher *r* to *z* transformation), then the results have been compared taking into consideration the sample size (Cohen and Cohen, 1983).

^4^Note that correcting for multiple comparisons is not necessary in this context. We reported the corrected values to show that even with a more conservative approach, the semantic of the pattern of results does not change.
